# Older adults with dual sensory loss in rehabilitation show high functioning and may fare better than those with single sensory loss

**DOI:** 10.1371/journal.pone.0237152

**Published:** 2020-08-03

**Authors:** Andrea Urqueta Alfaro, Dawn M. Guthrie, Cathy McGraw, Walter Wittich

**Affiliations:** 1 School of Optometry, University of Montréal, Montréal, Quebec, Canada; 2 Centre de Recherche Interdisciplinaire en Réadaptation du Montréal Métropolitain, Montreal, Canada; 3 Department of Kinesiology & Physical Education, Wilfrid Laurier University, Waterloo, Ontario, Canada; 4 Department of Health Sciences, Wilfrid Laurier University, Waterloo, Ontario, Canada; 5 CRIR/Lethbridge-Layton-Mackay Rehabilitation Centre of West-Central Montreal, Montréal, Quebec, Canada; 6 CRIR/Institut Nazareth et Louis-Braille du CISSS de la Montérégie-Centre, Montréal, Quebec, Canada; University of New South Wales, AUSTRALIA

## Abstract

The population of older adults that have Dual Sensory Loss (DSL) is increasing, yet most research to date has focused on single sensory impairment and is inconclusive as to whether DSL is associated with worse impact on health and well-being over single sensory loss. The primary aim of this study was to characterize the health and functioning of community-dwelling older adults with DSL who were receiving sensory rehabilitation, using an understudied assessment: the interRAI Community Health Assessment (CHA). The secondary aim was to investigate whether older adults with DSL had worse health-related outcomes than their peers with only vision loss (VL) or only hearing loss (HL). We report and compare the interRAI CHA results in a sample of 200 older adults (61+ years of age) who had DSL, VL or HL. Overall, all sensory impairment groups showed high functioning in the areas of cognition, communication, activities of daily living, depression, and psycho-social well-being. DSL was not always associated with worse outcomes compared to a single sensory loss. Rather, the results varied depending on the tasks assessed, as well as which groups were compared. Our findings highlight that despite the negative impact of sensory losses, community-dwelling older adults receiving sensory rehabilitation services tend to have overall good health and a high level of independence. These results also show that DSL is not always associated with worse outcomes compared to a single sensory loss. Further research is needed to better characterize older adults with DSL who have more severe sensory and cognitive difficulties than those in our sample, and among those who are not receiving rehabilitation services.

## Background

Researchers have traditionally investigated vision loss (VL) and hearing loss (HL) separately, but evidence suggests that these sensory impairments may be associated. In older adults (65+), as visual acuity worsens, the prevalence of HL increases [1], and VL and HL are often present concurrently, irrespective of age or order of onset of the sensory losses; a condition named dual sensory loss (DSL) [2]. DSL diminishes a person’s capacity to communicate, acquire information, perform daily activities, and fully participate in social environments without assistance or support (e.g., sign-language interpreter) [3–5]. Persons with DSL are at higher risk for numerous health issues including cognitive impairment, social isolation, impaired mobility, and depression [2,3,6]. Furthermore, persons with DSL are more likely to be unemployed, have low socio-economic status and poorer educational outcomes, compared to persons with other disabilities [7].

The prevalence of DSL is higher in older adults. Across four countries (Canada, US, Finland and Belgium), estimates ranged between 10% and 34% in older adults living in long-term care facilities, and between 13.4% and 24.6% in those receiving home care [6]. In older populations with higher levels of health needs (e.g., attending vision rehabilitation), the prevalence of DSL can be as high as 39% [2]. The prevalence of DSL is expected to increase because the population of most developed countries are aging [8]. Furthermore, the number of older adults aged 85 years or more is increasing more than in any other age group, and these individuals are the most likely to experience DSL and its associated challenges [9,10]. In the USA, it is projected that the population aged 85+ will experience a 129% increase, from 6.4 million in 2016 to 14.6 million by 2040 [8], and that by 2030, between 3.5 million and 14 million adults aged 70+ will develop DSL [11].

Despite the increasing numbers of older adults with DSL, most studies have focused on VL or HL alone than on DSL [1,11]. It is unclear whether DSL is associated with greater psychosocial impact over single sensory impairment because most prior studies have compared DSL only to no sensory impairment or to a comparison group that included both single and no sensory impairment [2,12,13]. Two alternative conceptual models can be used to understand the impact of DSL vis-á-vis single sensory impairment [12,14–16]. In one model, the impact results from the addition of the consequences of VL and HL. In the other model, there is an interaction between the effects of each single sensory impairment which multiplies the impact of DSL beyond what would result from merely adding such effects. According to the second model, a person may not be able to compensate for the loss in one sense by using the other sense. This becomes evident if we consider how DSL negatively affects the two primary modes of sensory communication; HL causes difficulties with listening whilst VL limits speechreading [11]. Making matters worse, most technological devices are designed to compensate for a single sensory impairment (e.g., closed-captioning, flashing alarm clock), limiting their usefulness for persons with DSL [11,17,18]. In both models, the impact of DSL would be larger than that of single sensory impairment. Although this seems reasonable research has not unequivocally supported this conclusion. One literature review of publications from 1996 to 2010 found inconclusive results as to whether DSL presents larger impacts compared to single sensory impairment [2]. A 2012 review suggested that the psychosocial and functional effects of DSL often, but not always, are larger than those of single sensory impairment, and that the mixed results likely depend of the type of task measured [19]. More recent research reports mixed results in the areas of physical, cognitive and mental health [20,21]. For instance, it is unclear whether DSL is associated with a greater risk of mortality compared to single sensory impairment. Some findings indicate that DSL (but not VL or HL) [22,23] shows greater risk whereas other results show that DSL and HL (but not VL) are associated with increased all-cause mortality [24].

In order to further exemplify the inconclusive findings with respect to the putative larger effect of DSL over single sensory impairment, in what follows we will review research on activities of daily living and depression. Instrumental activities of daily living (IADLs) involves performing complex tasks (i.e., higher cognitive and sensorimotor demands) like paying bills, and activities of daily living (ADLs) refer to less complex self-care personal tasks like dressing and bathing [25,26]. While a literature review showed that most evidence points to DSL being associated with greater ADL difficulties, compared to single impairment [26], other studies have found that results vary depending on the specific ADL task, and to which single sensory impairment DSL is compared to. For instance, DSL was shown to significantly increase the risk of difficulty with dressing and getting out of bed, only when compared to HL (but not VL), and having DSL did not increase the risk of difficulty with bathing, compared to either VL or HL [26]. Findings are also mixed with respect to IADL. In cross-sectional research, one study found that DSL is associated with greater difficulties with IADL compared to VL and HL, but that this effect disappears when scores are adjusted for age [27]. However, a second study showed that, after controlling for sociodemographic and physical health covariates, higher levels of DSL are associated with an increased risk of difficulties in some IADL tasks only when compared to VL (HL was not associated with higher functional disability) [26]. Yet a third cross-sectional study reported that DSL and VL have more limitations in IADL compared to HL [28], a finding that was also reported at baseline in a longitudinal study [29]. Moreover, the increase in difficulties in IADL with age differs depending on the specific IADL and the type of sensory loss. For instance, whereas issues with traveling and shopping increase the most for HL, followed by DSL, and then VL, difficulties preparing a meal increase the most for DSL, then HL, and lastly VL [29].

Depression is more common in older adults that have greater medical complexities [30] and in those with greater physical disability [31]. Evidence shows that DSL is associated with increased depressive symptoms or risk for depression [32,33], for instance longitudinal data indicates that the prevalence of depression increases faster in older adults that develop DSL versus those who do not [34]. These findings suggest that older adults with DSL would have worst levels of depression than those with single sensory impairment. However, results on this topic are mixed and cross-sectional studies report diverse patterns: no differences in number of depressive symptoms [35] or major depression [36] when comparing older adults with DSL versus single sensory impairment; DSL is associated with a higher likelihood of symptoms of depression compared to HL (but not VL) [37]; DSL is associated with a greater chance or rate of depression compared to HL and VL [38–40]. Findings from longitudinal research are also inconclusive. One study found that compared to older adults who did not develop sensory impairment, those that acquired DSL had the larger increase in depressive symptoms, approximately 1.5 times the increase associated with developing HL and 2 times with respect to VL [41]. However, other studies suggest different conclusions: DSL and VL (but not HL) are associated with more severe depression symptoms [12]; DSL and HL (but not VL) are associated with higher levels of depressive symptoms [42]; VL but not DSL are associated with depression [43].

The inconclusive results of research comparing the impact of DSL versus single sensory impairment may be due to differences among studies with respect to their samples and measures of sensory loss and health related outcome. For instance, the studies on depression reviewed above differed on their sample’s age inclusion criteria (e.g., 55+, 65+, 75+) and on how sensory loss was assessed, with some using objective measures (e.g., Snellen eye charts, pure-tone average) and others using self-report. Even in the latter case studies differed with respect to the specific questions asked of participants regarding their sensory functioning. Furthermore, studies used different measures of depression (e.g., Geriatric Depression Scale, DSM IV) and operationalized depression in diverse ways (e.g., symptoms of depression, major depression, risk for depression, the severity of the depressive symptoms). Additionally, evidence that worse outcomes in communication, ADL, and IADL are more prevalent in participants with the higher levels of sensory impairment [13] suggest that mixed findings may also result from samples that differ with respect to sensory impairment levels (i.e., mild, moderate, severe). Indeed, it has been proposed that the mixed findings with respect to the association of HL and all-cause mortality may be due to the fact that studies that did not find a significant association included participants with mild HL whereas a study that did find an association included participants with moderate to severe HL [24].

Several factors have been proposed as contributors to the limited research on DSL, including the tendency to focus on a single disability without considering the compounding effects of co-morbidity [11]. Despite these challenges, research is needed to characterize the population of older adults with DSL. The functioning of a person with sensory impairment is not solely determined by performance-based sensory assessments (e.g., visual acuity, pure-tone audiogram), but also by several other factors. For instance, physical and mental health are stronger predictors of quality of life in adults with VL, than contrast sensitivity, visual acuity, and the use of magnifiers [44]. Therefore, research is needed that aims to characterize older adults with DSL across a variety of domains relevant to their health and well-being. This is relevant for several reasons: policy makers can use this information to design and budget for intervention services; practitioners can utilize findings to tailor their interventions more appropriately; and the public can use this knowledge to better understand the challenges facing older adults with DSL.

Assessments developed by the interRAI (http://www.interrai.org/), a not-for-profit research network, are currently being used internationally to assess the health and well-being of older adults including those with sensory impairment [45–47]. However, there is limited research that has used interRAI assessments to characterizes older adults with DSL. Furthermore, studies have focused on older adults who were receiving home care services or living in residential care, many of whom present multiple factors that impact their physical and cognitive functioning including multiple co-morbid health conditions and chronic illnesses [13,48]. Prior research with older adults in home care indicates that compared to participants without DSL, those with DSL are more likely to have difficulty with communication, impaired cognitive performance, compromised independence with ADLs and IADLs, and increased risk of depression [6,13]. Similarly, older adults with DSL living in residential care are more likely to experience moderate to severe cognitive impairment, severe impairment in ADLs and higher rates of communication difficulties, compared to peers without DSL [6]. Unfortunately, the comparison groups included in these studies grouped together no sensory impairment and single sensory impairment, and thus the impact of DSL vis-á-vis that of single sensory impairment could not be investigated. Furthermore, the findings from this prior research have limited generalizability to older adults with DSL living in the community who are likely to show lower levels of need for assistance and greater capability to participate in sensory rehabilitation. Of the interRAI instruments, the Community Health Assessment (CHA) is best suited to assess sensory impairment, health, functioning and well-being of community-dwelling older adults who have DSL or single sensory impairment [49]. To our knowledge only one prior study used the interRAI CHA in these older adults however its focus was on the assessment’s sensitivity and specificity rather than on a comprehensive report of the sample’s assessment results [49].

The primary aim of the present study was to contribute to the comprehensive characterization of older adults with DSL by reporting the results of the interRAI CHA in a sample of community-dwelling older adults with DSL, HL, and VL who were receiving sensory rehabilitation services. The data were originally collected for a study that assessed how well the interRAI CHA identified older adults with DSL, in comparison to performance-based measurements of hearing and vision loss [49]. The comprehensive nature of the interRAI CHA (see methods) allows the sample to be characterized in terms of several factors, including demographic characteristics, cognitive performance, communication, depression, ADL, and performance in activities that require vision or hearing (“functional” vision or hearing). A secondary aim of this study was to investigate whether older adults with DSL have worse health-related outcomes than their peers with single sensory impairment.

## Methods

This study' procedures were reviewed and approved by the *Centre de recherche interdisciplinaire en réadaptation de Montréal métropolitain* (CRIR-1018-1114). All investigations were performed according to the guidelines of the Declaration of Helsinki and all participants gave informed written consent [50]. This paper followed the STrengthening the Reporting of OBservational studies in Epidemiology (STROBE) guidelines [51] for accurate and complete reporting of an observational study. The detailed methodology has been reported elsewhere [49]; here, we present a summary of the most relevant methodological aspects.

### Participants

Participants were recruited among older adults (61 years old or older) that were attending sensory rehabilitation centres in Montreal and were eligible for sensory rehabilitation services as defined by the Quebec Ministry of Health (i.e., a visual acuity in the better eye with best standard correction of 20/60 (6/18) or less, or a visual field diameter of < 60 degrees in the better eye, or hemianopsia (loss of half of the visual field); and/or an unaided pure-tone average decibel hearing loss (dB HL) in the better ear of 35 dB HL or more across 4 frequencies: 0.2, 1, 2, and 4 kHz [52,53]). Recruitment and data collection took place from August of 2015 to July of 2017. starting at least 6 months before study measures were collected. Starting at least 6 months before study measures were collected, all participants received a variety of individualized services that are offered within the Quebec health system at no charge to the client [54].

### Measures

#### interRAI Community Health Assessment (CHA)

The interRAI CHA is a standardized clinical assessment that assesses the overall health and functional abilities of adults (18 years of age or older), and guides an assessor in the development of a service plan [55]. The interRAI CHA consists of roughly 150 items that capture the respondent’s basic demographics and detailed information across 13 domains (e.g., activities of daily living, cognition, mental health). The interRAI CHA has a Deafblind Supplement (DbS) which includes 150 additional items that gather information across 11 domains considered relevant for the assessment of an individual with DSL (e.g., vision, hearing, communication ability) [56]. The responses to all items within the interRAI CHA and DbS are closed-ended. For completing the assessment, assessors use their clinical impressions and information from multiple sources (e.g., respondent’s self-report, caregivers and health professional reports, medical records). The assessor does not test sensory function using objective measures (i.e., acuity test, audiogram), but if he has access to medical records that include results from these type of tests, he uses this information when completing the assessment. Participants completed the interRAI CHA using their sensory devices (i.e., corrective eyeglasses, hearing aids). To facilitate communication the assessor used effective communication strategies [57], and a personal amplification device (Pocket Talker®) was made available to participants who required an assistive listening device. Research shows that the interRAI CHA, DbS, and associated scales, have good internal consistency, convergent validity [56,58], and acceptable inter-rater reliability [59]. They are the only standardized assessment instruments we are aware of for identifying the needs, strengths and challenges of adults with DSL.

In this study we included all six health index scales that are generated directly from items within the interRAI CHA and DbS, capturing domains such as activities of daily living, cognitive performance, and depressive symptoms. On all scales, a higher score indicates a greater level of impairment/difficulty [6]. For instance, the Deafblind Severity Index (DbSI) identifies persons with DSL (score of 3 indicating vision and hearing are mild/moderately impaired). Research supports the concurrent validity of the DbSI, such that a higher score is associated with greater difficulties interacting with others and performing instrumental activities of daily living [56]. We report the sample’s health index scale scores divided into groups representing increasing levels of impairment/difficulty. The only exception to this is the DbSI, in which case we report the number of participants than scored 3+ that is, that qualified as having DSL. Information about all health index scales, including how scores were grouped, is provided in S1 Table.

We also generated all eleven clinical assessment protocols (CAPs). Based on an algorithm represented by several items in the assessment, a CAP can be “triggered”, indicating that the respondent has a current issue or is at risk for having an issue in the future with respect to the area assessed by the CAP [60]. For instance, the Communication CAP aims to assesses the need for intervention to maintain or improve communication functioning; the Informal Support CAP identifies persons who require help with IADL. The outcome of a CAP consists of categories that indicate whether the person is at risk for an issue (i.e., “not triggered”, “triggered”), and how significant is the risk (e.g., “medium risk”, “high risk”). The sample’s CAP results are reported using the CAP’s original outcome categories, that is, they were not grouped into new strata. See S1 Table for details on all the CAPs included in this study.

Additionally, we report the results of several interRAI CHA and DbS items. These items provide information about the sample’s: demographics (e.g., age, residential status); sensory impairment characteristics (e.g., age at onset of vision or hearing loss, difficulty with vision or hearing); cognition (e.g., daily decision making); communication (e.g., comprehension); psychological well-being (e.g., participation in social activities of long-standing interest, length of time alone during the day); and co-morbidities (the count of co-morbidities was calculated from a list of 14 diseases, i.e., hip fracture, other fracture, Alzheimer’s disease, dementia other than Alzheimer’s disease, stroke/ cerebrovascular accident, coronary heart disease, chronic obstructive pulmonary disease, congestive heart failure, anxiety, bipolar disorder, depression, schizophrenia, cancer, diabetes mellitus, other disease diagnoses). Details of the response options for these items are included in Tables 1 through 5.

**Table 1:** Demographic characteristics of participants.

	Vision loss only	Hearing loss only	Dual sensory loss	
	N = 58	N = 69	N = 73	
Demographic characteristic	% (N)			p-value
*Sex*				
Female	60.3 (35)	55.1 (38)	67.1 (49)	0.54
Male	39.7 (23)	44.9 (31)	32.9 (24)	
*Age (mean and sd*^*a*^*)*	79.9 (8.2)	78.6 (7.3)	85.0 (8.3)	
61–74	31.0 (18)	29.0 (20)	15.1 (11)	0.007
75–84	34.5 (20)	44.9 (31)	23.3 (17)	
85+	34.5 (20)	26.1 (18)	61.6 (45)	
*Education*				
Less than high school	19 (11)	8.7 (6)	24.7 (18)	0.21
Some high school	15.5 (9)	23.2 (16)	15.1 (11)	
High school or trade school	31.0 (18)	18.8 (13)	26.0 (19)	
Post-secondary	34.5 (20)	49.3 (34)	34.2 (25)	
*Marital status*				
Never married	13.8 (8)	5.8 (4)	4.1 (3)	
Married/partner/significant other	43.1 (25)	42.0 (29)	28.8 (21)	0.12
Widowed/separated/divorced	43.1 (25)	52.2 (36)	67.1 (49)	
*Living Arrangement*				
Alone	48.3 (28)	44.9 (31)	60.3 (44)	0.49
With spouse/partner only	39.7 (23)	43.5 (30)	30.1 (22)	
With spouse/partner and other(s)	3.4 (2)	1.4 (1)	1.4 (1)	
With child (no spouse/partner)	34. (2)	7.2 (5)	5.5 (4)	
With sibling(s)	0.0	0.0	2.7 (2)	
With nonrelative(s)	5.2 (3)	2.9 (2)	0.0	
*Residential Status*				
Private home/apartment/rented room	81.0 (47)	91.3 (63)	72.6 (53)	0.07
Assisted living or semi-independent living	17.2 (10)	7.2 (5)	26.0 (19)	
Other	1.7 (1)	1.4 (1)	1.4 (1)	
*Primary language*				
English	67.2 (39)	68.1 (47)	60.3 (44)	0.75
French	32.8 (19)	31.9 (22)	39.7 (29)	

^a^sd = standard deviation, N = number.

**Table 2:** Visual impairment characteristics comparing individuals with VL versus those with DSL.

	Vision loss only	Dual sensory impairment	
	N = 58	N = 73	
Vision impairment characteristic	% (N)		p-value
*Vision diagnosis*^*a*^			
Macular Degeneration	55.2 (32)	72.6 (53)	0.09
Diabetic Retinopathy	8.6 (5)	5.5 (4)	0.73
Retinitis Pigmentosa	6.9 (4)	8.2 (6)	1.00
Other retinal conditions	20.7 (12)	6.9 (5)	0.04
Glaucoma	24.1 (14)	28.8 (21)	0.75
Pseudoaphakia	19 (11)	20.5 (15)	0.86
Cataracts	10.3 (6)	11.0 (8)	0.92
*Age at onset of vision loss*			
0–2 years	5.2 (3)	2.7 (2)	0.69
3–18 years	1.7 (1)	0.0	
19–64 years	25.9 (15)	20.5 (15)	
65+ years	67.2 (39)	76.7 (56)	
*Near vision (with vision device if applicable)*			
Adequate	0	0	0.37
Minimal difficulty	3.4 (2)	13.7 (10)	
Moderate difficulty	91.4 (53)	79.5 (58)	
Severe difficulty	3.4 (2)	4.1 (3)	
No vision	1.7 (1)	4.1 (3)	
*Distance vision (with vision device if applicable)*			
Adequate	1.7 (1)	2.7 (2)	0.37
Minimal difficulty	8.6 (5)	6.8 (5)	
Moderate difficulty	72.4 (42)	83.6 (61)	
Severe difficulty	13.8 (8)	2.7 (2)	
No vision	3.4 (2)	4.1 (3)	
*Visual field diameter (without low vision devices)*			
Normal or near normal vision: > 80 degrees	63.8 (37)	54.8 (40)	0.74
Moderate low vision: 40–80 degrees	15.5 (9)	21.9 (16)	
Severe low vision: 16 degrees to < 40 degrees	3.4 (2)	4.1 (3)	
Profound low vision: 8 degrees to < 16 degrees	0.0	1.4 (1)	
Near-blindness: 4 degrees to < 8 degrees	3.4 (2)	0.0	
Blindness: no visual field to < 4 degrees	6.9 (4)	5.5 (4)	
Unknown	6.9 (4)	12.3 (9)	
*Difficulties in adverse seeing conditions*			
Dim light or at night	53.4 (31)	64.4 (47)	0.41
Bright light or daylight	56.9 (33)	45.2% (33)	0.37
Glare or stray light	87.9 (51)	90.4 (66)	0.81
Abrupt changes in illumination	77.6 (45)	68.5 (50)	0.46
Subtle contrast differences	17.2 (10)	35.6 (26)	0.07
*Use of sight substitution devices in the last 3 days*			
No	51.7 (30)	35.6 (26)	0.16
Yes	48.3 (28)	64.4 (47)	
*Use of Orientation and Mobility devices in the last 3 days*			
No	44.8 (26)	35.6 (26)	0.49
Yes	55.2 (32)	64.4 (47)	

^**a**^ **Participants could report more than one vision diagnosis.**

**Table 3:** Hearing impairment characteristics comparing individuals with HL versus those with DSL.

	Hearing loss only	Dual sensory impairment	
	N = 69	N = 73	
Hearing impairment characteristics	(%) N		p-value
*Main hearing diagnosis*^*a*^			
Presbycusis	73.9 (51)	63.0 (46)	0.37
Tinnitus or head/ear noise	8.7 (6)	5.5 (4)	0.74
Congenital Syndromes	4.4 (3)	6.9 (5)	0.83
Unknown	15.9 (11)	17.8 (13)	0.85
*Hearing loss onset age*			
0–2 years	2.9 (2)	0.0	0.001
3–18 years	8.7 (6)	1.4 (1)	
19–64 years	34.8 (24)	12.3 (9)	
65+ years	53.6 (37)	76.7 (56)	
*Hearing (with hearing device if applicable)*			
Adequate	0.0	0.0	0.04
Minimal difficulty	58.0 (40)	80.8 (59)	
Moderate difficulty	34.8 (24)	17.8 (13)	
Severe difficulty	7.2 (5)	1.4 (1)	
*Alerting to voices at normal volume*			
Responds without hearing devices	5.8 (4)	17.8 (13)	0.07
Responds with hearing devices	91.3 (63)	82.2 (60)	
Does not respond	2.9 (2)	0.0	
*Use of hearing devices in the last 3 days*			
No	14.5 (10)	20.5 (15)	0.54
Yes	85.5 (59)	79.5 (58)	

^a^ Participants could report more than one hearing diagnosis.

**Table 4:** Health and well-being comparing the three sensory impairment groups.

	Vision loss only	Hearing loss only	Dual sensory impairment	
	N = 58	N = 69	N = 73	
Item or Health Index Scale	% (N)			p-value
**Cognition**				
*Change in decision making (last 90 days) item*				
Improved (0)	1.7 (1)	1.4 (1)	0 (0)	0.23
No change (1)	93.1 (54)	97.1 (67)	90.4 (66)	
Declined (2)	3.4 (2)	1.4 (1)	9.6 (7)	
Uncertain (8)	1.7 (1)	0.0	0.0	
*Daily decision making*				
Independent (0)	96.6 (56)	98.6 (68)	98.6 (72)	0.86
Modified independence (1)	1.7 (1)	1.4 (1)	1.4 (1)	
Minimally impaired (2)	1.7 (1)	0.0	0.0	
*Memory/recall ability*				
No memory problem (0)	93.1 (54)	97.1 (67)	94.5 (69)	0.75
Memory problem (1)	6.9 (4)	2.9 (2)	5.5 (4)	
*Cognitive Performance Scale (CPS)*				
Intact (0)	91.4 (53)	95.7 (66)	94.5 (69)	0.77
Borderline intact (1) or mild impairment (2)	8.6 (5)	4.4 (3)	5.5 (4)	
**Communication**				
*Communication comprehension*				
Understands others (0)	96.6 (56)	20.3 (14)	41.1 (30)	0.001
Usually understands others (1)	3.4 (2)	65.2 (45)	52.1 (38)	
Often understands others (2)	0.0	14.5 (10)	6.8 (5)	
*Expressive communication (ability to be understood by others)*				
Understood (0)	98.3 (57)	98.6 (68)	98.6 (72)	0.85
Usually understood (1)	1.7 (1)	0.0	1.4 (1)	
Rarely or never understood	0.0	1.4 (1)	0.0	
**Activities of Daily Living (ADL)**				
*ADL Self-performance Hierarchy Scale*				
Independent (0)	91.4 (53)	98.6 (68)	93.2 (68)	0.54
Supervision required (1) or limited impairment (2)	5.2 (3)	1.5 (1)	2.7 (2)	
Extensive assistance required– 1 (3) or extensive assistance required—2 (4)	3.5 (2)	0 (0)	4.1 (3)	
*IADL Capacity Scale*				
Independent (0),	46.6 (27)	70.0 (48)	34.3 (25)	0.002
Set-up help only (1) or supervision (2)	16.9 (9)	14.5 (10)	33.0 (24)	
Limited assistance (3) or extensive assistance (4)	38.0 (22)	16.0 (11)	33.0 (24)	
**Psycho-social Well-being**				
*Participation in social activities of long-standing interest*				
Never (0)	3.5 (2)	1.5 (1)	1.4 (1)	0.85
More than 30 days ago (1)	3.5 (2)	8.7 (6)	4.1 (3)	
8 to 30 days ago (2)	6.9 (4)	10.1 (7)	6.9 (5)	
4 to 7 days ago (3)	20.7 (12)	24.6 (17)	30.1 (22)	
Within the last 3 days (4)	65.5 (38)	55.1 (38)	57.5 (42)	
*Visit with a long-standing social relation or family member*				
Never (0)	3.5 (2)	1.5 (1)	1.4 (1)	0.62
More than 30 days ago (1)	5.2 (3)	0 (0)	1.4 (1)	
8 to 30 days ago (2)	12.1 (7)	8.7 (6)	13.7 (10)	
4 to 7 days ago (3)	25.9 (15)	21.7 (15)	30.1 (22)	
Within the last 3 days (4)	53.5 (31)	68.1 (47)	53.4 (39)	
*Other interaction with long-standing social relation or family member*				
Never (0)	3.5 (2)	1.5 (1)	1.4 (1)	0.82
More than 30 days ago (1)	3.5 (2)	0 (0)	1.4 (1)	
8 to 30 days ago (2)	0 (0)	1.5 (1)	1.4 (1)	
4 to 7 days ago (3)	19.0 (11)	12.0 (8)	15.1 (11)	
Within the last 3 days (4)	74.1 (43)	85.5 (59)	81.0 (59)	
*Conflict or anger with family or friends*				
Never (0)	15.5 (9)	4.4 (3)	12.3 (9)	0.04^a^
More than 30 days ago (1)	72.4 (42)	90.0 (62)	82.2 (60)	
8 to 30 days ago (2)	0 (0)	0 (0)	4.1 (3)	
4 to 7 days ago (3)	1.7 (1)	1.5 (1)	1.4 (1)	
Within the last 3 days (4)	8.6 (5)	4.4 (3)	0 (0)	
Unable to determine	1.7 (1)	0 (0)	0 (0)	
*Fearful of a family member or close acquaintance*				
Never (0)	93.1 (54)	98.6 (68)	100 (73)	0.07
More than 30 days ago (1)	6.9 (4)	1.5 (1)	0 (0)	
*Neglected*, *abused*, *or mistreated *				0.09
Never (0)	91.4 (53)	94.2 (65)	100 (73)	
More than 30 days ago (1)	6.9 (4)	5.8 (4)	0 (0)	
8 to 30 days ago (2)	1.7 (1)	0 (0)	0 (0)	
*The person or others state that he/she comments on feeling lonely (even if visited regularly)*				
No (0)	82.8 (48)	87.0 (60)	87.7 (64)	0.82
Yes (1)	17.2 (10)	13.0 (9)	12.3 (9)	
*Change in social activities in last 90 days*: *changes in the quantity*, *frequency and quality of social activities*. *Distress refers to the person’s mood being affected by a change in participation*				
No decline (0)	79.3 (46)	81.2 (56)	82.2 (60)	0.86
Decline, not distressed (1)	15.5 (9)	14.5 (10)	16.4 (12)	
Decline, distressed (2)	5.2 (30	4.4 (3)	1.4 (1)	
*Amount of time the person is literally alone or spends by him- or herself in his or her own room (if living with other people)*				
Less than 1 hour (0)	15.5 (9)	13.0 (9)	12.3 (9)	0.42
1–2 hours (1)	36.2 (21)	45.0 (31)	27.4 (20)	
More than 2 hours but less than 8 hours (2)	39.7 (23)	29.0 (20)	50.7 (37)	
8 hours or more (3)	8.6 (5)	13.0 (9)	9.6 (7)	
*Major life stressors in last 90 days*: *experiences that disrupted or threatened to disrupt a person’s daily routine and that imposed readjustment*				
No (0)	67.2 (39)	78.3 (54)	86.3 (63)	0.09
Yes (1)	32.8 (19)	21.7 (15)	13.7 (10)	
*Depression Rating Scale (DRS)*				
No signs/symptoms of depression (DRS score of 0–2)	93.1 (54)	100 (69)	100 (73)	0.03
Signs/symptoms of depression (DRS scores of 3+)	6.9 (4)	0 (0)	0 (0)	
**Comorbidities**				
None	21.0 (12)	13.0 (9)	15.1 (11)	0.54
1	36.2 (21)	26.1 (18)	34.3 (25)	
2	17.2 (10)	32.0 (22)	32.0 (23)	
3 or more	26.0 (15)	29.0 (20)	19.2 (14)	
**Pain Scale**				
No pain (0)	37.9 (22)	40.6 (28)	42.5 (31)	0.77
Less than daily pain (1), daily not severe pain (2)	53.5 (31)	55.1 (38)	46.6 (34)	
Daily severe pain (3), daily excruciating pain (4)	8.6 (5)	4.4 (3)	11.0 (8)	
**Use of adaptive devices item in last 3 days (e.g., specialized lighting, TDD)**				
No	5.2 (3)	34.8 (24)	0.0	0.001
Yes	94.8 (55)	65.2 (45)	100 (73)	

IADL = Instrumental Activities of Daily Living.

^a^ No association after controlling for age (χ^2^ (10, N = 200) = 16.15, p = 0.09), cognition (χ^2^ (10, N = 200) = 17.53, p = 0.06), risk of depression (χ^2^ (10, N = 200) = 14.58, p = 0.14), comorbidities (χ^2^ (10, N = 200) = 17.58, p = 0.06).

**Table 5:** Triggering on the Clinical Assessment Protocols (CAP) by sensory impairment group.

	Vision loss only	Hearing loss only	Dual sensory impairment	
	N = 58	N = 69	N = 73	
CAP	% (N)			p-value
*Cognitive CAP*				
Not triggered (0)	98.3 (57)	98.6 (68)	100 (73)	0.36
Triggered–prevent decline (1)	1.7 (1)	1.5 (1)	0 (0)	
*Communication CAP*				
Not triggered (0)	96.6 (56)	98.6 (68)	98.6 (72)	0.86
Triggered—potential for improvement (1)	1.7 (1)	1.5 (1)	1.4 (1)	
Triggered—risk of decline (2)	1.7 (1)	0 (0)	0 (0)	
*Informal Support CAP*				
Not triggered (0)	75.9 (44)	87.0 (60)	56.2 (41)	0.007
Triggered (1)	24.1 (14)	13.0 (9)	43.8 (32)	
*Instrumental Activities of Daily Living CAP*				
Not triggered (0)	93.1 (54)	97.1 (2)	89.0 (65)	0.37
Triggered–with potential to improve (1)	6.9 (4)	2.9 (2)	11.0 (8)	
*Mood CAP*				
Not triggered (0)	69.0 (40)	84.1 (58)	86.3 (63)	0.07
Triggered—medium risk (1)	24.1 (14)	15.9 (11)	13.7 (10)	
Triggered—high risk (2)	6.9 (4)	0 (0)	0 (0)	
*Prevention CAP*				
Not triggered (0)	0 (0)	26.1 (18)	8.2 (6)	0.001
Triggered–had physician visit (1)	69.0 (40)	45.0 (31)	60.3 (44)	
Triggered–no physician visit (2)	31.0 (18)	29.0 (20)	31.5 (23)	
*Social Relationship CAP*				
Not triggered (0)	82.8 (48)	87.0 (60)	88.0 (64)	0.82
Triggered for care plan follow Up (1)	17.2 (10)	13.0 (9)	12.3 (9)	
*Dual Sensory Loss (DSL) Orientation and Mobility (O&M) CAP*				
Not triggered (0)	N/A	N/A	8.7 (6)	N/A
Triggered–DSL & difficulty with O&M in unfamiliar environments (1)	N/A	N/A	84.1 (58)	
Triggered–DSL & difficulty with O&M in familiar environments (2)	N/A	N/A	7.3 (5)	

### Data analysis

The classification of participants into sensory impairment groups following previously established interRAI CHA standards. Participants were identified as having DSL if their DbSI score was 3+. They were classified as having VL only if their result on the interRAI CHA item on near vision (using vision device if applicable) indicated at least minimal difficulty (score = 1 or higher) and their result on the item on hearing indicated no difficulty (score = 0). If the inverse was the case, at least minimal difficulty with hearing and no difficulty with near vision, participants were classified as having HL only. Thus, the interRAI CHA identification of VL did not include issues with distance vision or contrast sensitivity.

For all data analyses described in the following, we utilized the chi-square test or Fisher’s exact test, if the chi-square test assumptions were not met. To account for the large number of tests of association being made, all *P* values were adjusted using the false discovery rate control [61]. The adjusted *P* values were based on the number of tests completed and the false discovery rate. A statistically significant difference was defined using a two-tailed alpha level of 0.05.

To investigate whether older adults with DSL had worse assessment results than those with single sensory impairments, we conducted tests of association between the three sensory impairment groups and items on demographics (e.g., age, highest educational level). We also explored if there were significant associations between the sensory impairment group VL or DSL and items related to visual impairment (e.g., age at VL onset, visual difficulty in adverse seeing conditions). Similar analyses were carried out to compare the HL and DSL groups across items related to hearing impairment. These analyses were necessary to identify factors other than the number of senses impaired (e.g., older age, unequal sensory impairment characteristics) that could explain significant differences among the groups’ assessment results.

To compare the assessment outcomes of the three sensory impairment groups, we conducted tests of associations between the groups and the results of the interRAI CHA and DbS items, all six health index scales, and all eleven CAPs.

We also investigated whether the results of the interRAI CHA health index scales and the CAPs differed significantly between men and women.

We explored associations between sensory impairment groups and assessment outcomes after controlling for the following covariates: age (stratified into 3 groups: 61–74, 75–84, 85+ years), cognition (as measured with the Cognitive Performance Scale with scores divided into two levels: cognitively intact versus borderline intact or mild impairment), risk of depression (as measured with the MOOD CAP which has three possible outcome: not triggered, triggered with medium risk of depression, triggered with high risk of depression), and number of comorbidities (0, 1, 2, 3+). We calculated the Cochran-Mantel-Haenszel chi-square value for each of the covariates. In the results section we report when an association was no longer significant after controlling for a covariate.

## Results

### Sensory impairment classification

Based on the DbSI score, 36.5% (N = 73) of the participants had DSL; of these 91.8% (N = 67) had both mild/moderately impaired vision and hearing; and 8.2% (N = 6) had one of these senses mild/moderately impaired, while the other was severely impaired. Based on the results on the items on vision and hearing, 29.0% (N = 58) of the participants had VL only, and 34.5% (N = 69) had HL only. See section on Sensory Impairment Characteristics for further information on levels of sensory difficulty.

### Demographic characteristics

The sensory impairment groups were only significantly different with respect to age in that a higher proportion of participants with DSL (61.6%, N = 45) were aged 85+ as compared to those with VL (34.5%, N = 20) and with HL (26.1%, N = 18), χ^2^ (4, N = 200) = 20.89, p = 0.007 (Table 1).

### Sensory impairment characteristics

#### Visual loss

Most variables related to the participants’ visual impairment characteristics were not significantly associated with sensory impairment group (VL or DSL). In both groups, most participants acquired VL at age 65+ years, reported moderate difficulties with near and distance vision, and had normal to near to normal visual field. A lower percentage of participants with DSL (6.9%, N = 5) were classified as having “other retinal conditions” (i.e., other than Macular Degeneration, Diabetic Retinopathy and Retinitis Pigmentosa), compared to those with VL (20.7%, N = 12, p = 0.04).

#### Hearing loss

Compared to participants with HL, in those with DSL a higher percentage had “minimal” difficulty with hearing using hearing device if applicable (80.8% vs. HL at 58.0%; Fisher’s p = 0.04), suggesting that participants with DSL had lower levels of hearing difficulty (Table 3). Additionally, a higher percentage of participants with DSL (76.7%) acquired HL at age 65+ (HL = 53.6%; Fisher’s p = 0.001.

### Health, functioning and well-being

In the area of cognition, the majority of participants in all sensory impairment groups were classified in categories indicating higher functioning, and groups did not differ significantly in their results. This was the case for the items: change in decision making in last 90 days, daily decision making and memory recall ability (Table 4). In all groups, over 90% of participants were classified as cognitively intact by the CPS Scale (Fisher’s p = 0.77, Table 4), and nearly all participants (98.3% - 100%) failed to trigger the Cognitive CAP (Fisher’s p = 0.36, Table 5).

With respect to expressive communication and comprehension (Table 4), most participants in all groups were classified in the higher functioning categories. Groups only significantly differed with respect to comprehension in that most participants with VL (96.6%) were classified as “understands others” (HL = 20.3%, DSL = 41.1%), whereas most of those with HL (65.2%) and DSL (52.1%) were classified as “*usually* understands others”, χ^2^ (4, N = 200) = 78.13, p = 0.001. In all groups, over 96% of participants did not trigger the Communication CAP, Fisher’s p = 0.86 (Table 5).

Most participants (91.4% to 98.6%; Fisher’s p = 0.54) were independent in completing their ADLs (Table 4). However, a lower percentage of participants with VL (46.6%) and DSL (34.3%) were classified as independent (HL = 70.0%; χ^2^ (4, N = 200) = 22.09, p = 0.002), with respect to their IADLs. Most participants, in all three groups, did not trigger the IADL CAP nor the Informal Support CAP (Table 5). In the case of the Informal Support CAP, a higher percentage of participants with DSL (43.8%) and VL (24.1%) triggered this CAP (HL = 13.0; χ^2^ (2, N = 200) = 17.33, p = 0.007).

In the areas of depression and psycho-social well-being, most participants in all sensory impairment groups scored on the positive end of the respective items (e.g., no risk of depression) (Tables 4 and 5). Only the VL group had participants (6.9%, N = 4) that scored 3+ in the Depression Rating Scale suggesting clinical depression [62,63], no participants with HL or DSL had such score (Fisher’s p = 0.03) (Table 4).

In the items regarding psycho-social well-being (Table 4), most participants in all sensory impairment groups were not experiencing issues such as loneliness and neglect. Only the item “conflict or anger with family or friends” was associated with sensory impairment group (Fisher’s p = 0.04). However, this association was no longer significant after controlling for age (χ^2^ (10, N = 200) = 16.15, p = 0.09), cognition (χ^2^ (10, N = 200) = 17.53, p = 0.06), and risk of depression (χ^2^ (10, N = 200) = 14.58, p = 0.14).

Most participants in all groups (82.8% to 87.7%) did not trigger the Social Relationships CAP (Table 5), indicating that they were not at risk of reduced social relationships and did not require intervention to facilitate social engagement, χ^2^ (2, N = 200) = 0.73, p = 0.82.

High functioning results were also found with respect to the CAPs Appropriate medication, Dehydration, Falls and Urinary incontinence which were not triggered in most participants in all sensory impairment groups. Results of the Pain CAP were more mixed with roughly 46% to 55% in all groups reporting less than daily or daily but not severe pain (S2 Table). Regarding the use of each type of assistive devices or supports (e.g., sight enhancement devices, hearing devices) between about 50 to 100% of participants in all sensory impairment groups reported usage (Tables 2, 3 and 4).

In contrast with the high functioning results reported thus far, the sample’s outcome in the Prevention, and O&M CAPs (Table 5) indicated lower outcomes and the need for intervention. The Prevention CAP was triggered for the vast majority of participants in all groups, and was associated with sensory impairment group with higher percentages of participants with VL (100%) and DSL (91.8%) triggering this CAP (HL = 74%), χ^2^ (4, N = 200) = 22.6421, p = 0.001. This suggests that having VL (alone or in combination with HL) was associated with poorer preventive health care, comparing to having HL alone. The O&M CAP, which only applies to participants with DSL, was triggered in 84.96% of participants since they had difficulties moving about in unfamiliar environments, and in an additional 7.3% due to difficulties moving about in familiar environments.

The only significant associations with sex were found for the Informal Support CAP, the Pain scale, and the item “length of time alone during the day”, with females showing poorer results (S3 Table).

## Discussion

In most of the areas assessed (cognition, communication, ADLs, IADLs, mental health, social relationships), the majority of participants in all sensory impairment groups showed high functioning and were not at risk for health problems in the immediate future, as measured with the interRAI CHA. Our findings highlight that despite having sensory losses, community-dwelling older adults who receive rehabilitation services are experiencing overall good health and a high level of independence.

These results are more positive than those reported previously in research with older adults that used other interRAI measurements, with respect to cognition [3,6,13], ADL, IADL [6,13], and symptoms of depression [6,13]. This comparison confirms our expectation that findings from these previous studies which investigated older adults receiving home care or living in residential care cannot be extended to older adults living in the community. As we expected, our sample of community-dwelling older adults show better health and functioning in several areas, compared to peers that receive home care or live in residential care. The high level of functioning in our sample is also likely in part a consequence of the recruitment through a rehabilitation service, as all participants had access to sensory devices, learning of compensatory strategies, and participation in social activities. Particularly with respect to depression, it has been proposed that rehabilitation services and the use of assistive devices can reduce depression in older adults with DSL [64]. Additionally, the use of hearing aids is associated with better cognitive performance in adults (40–69 years old) [65,66]. Our sample is particularly advantaged because in Quebec access to rehabilitation services and sensory assistive devices is publicly-funded and users incur no costs [54]. Indeed, our sample has an overall high level of sensory device (including hearing aids) use.

It is noteworthy that DSL was not always associated with worse results, compared to the single sensory impairment groups. In the areas of difficulty with IADL, the need of preventive health care, and the ability to live independently, having VL (alone or with concurrent HL) was associated with worse outcomes compared to having HL alone. Our IADL result contradicts a previous study which found that after controlling for age DSL was not associated with greater difficulties with IADL, compared to single sensory impairment [27]. This is so despite the fact that this prior study used the Lawton IADL scale which highly correlates with our IADL measure, the IADL capacity scale [67]. This prior study does not report the sample’s living arrangement and thus it is unknown if our diverse findings arise from differences in the sample’s living arrangement. Our finding do support previous evidence from samples of community-dwelling older adults which indicates that DSL and VL have worse IADL outcomes, compared to HL [28,29]. It is noteworthy that these prior studies used IADL measures that were different to ours. It has been suggested that differences in IADL outcomes among sensory impairment groups may arise from differences in the severity of their impairments [13]. However, the worse outcomes of our participants with DSL, compared to those with HL are not likely to arise from the DSL group having worse hearing functioning than the HL group. On the contrary, compared to those with HL, a higher percentage of participants with DSL had lower levels of hearing difficulty. Likewise, the difference is not likely due to participants in the DSL group having had more time to adapt to the hearing impairment as a higher percentage of participants with DSL acquired it at age 64+ years, compared to those with HL.

In line with the idea that whether DSL is associated with worse outcomes than single sensory impairment depends on the type of task measured [19], the better IADL functioning in our participants with HL alone may result in part from the diverse vision and hearing demands of the types of tasks assessed with the CHA. For instance, if we consider the IADL tasks, presumably only one of them (phone use) is more dependent on hearing, whereas the other two (housework, meal preparation) are more dependent on vision. In addition, it is reasonable that concurrent loses in vision and hearing would have a greater influence on a person’s ability to live independently, hence the higher percentage of individuals with DSL in our sample living in assisted or semi-independent living arrangements. However, after controlling for covariates (i.e., age, cognition, risk of depression, comorbidities) we found no significant difference among sensory impairments groups with respect to living arrangements. This result and that of others that found significant differences among sensory impairment groups to disappear after controlling for covariates [27] highlight the importance of conducting statistical analyses that accounts for the role of variables other than sensory impairment. It is noteworthy that the vast majority of our participants with DSL triggered the O&M CAP because they did not move independently in unfamiliar environments. This CAP is triggered when the participant has potential for improvement, therefore these results indicate an area of need that could be further addressed by rehabilitation services.

In line with the idea that differences between DSL and single sensory impairment vary depending on the tasks that are measured [19,26] we found different patterns of results across tasks. As we discussed earlier, with respect to IADL and the ability to live independently, having VL (alone or with concurrent HL) was associated with worse outcomes compared to having HL alone. In contrast, with regards to communication comprehension, having HL (alone or with concurrent VL) was associated with worse outcomes than having VL alone. Our results on communication comprehension make sense when we consider how each sensory impairment impacts comprehension. It is likely that HL results in more impairment than VL on a person’s comprehension of communication, which relies heavily on verbal expression. In addition, individuals with DSL have difficulties accessing compensatory visual means of communication (e.g., speech-reading) due to their impaired vision. Nonetheless, surprisingly, in our sample of older adults that have accessed rehabilitation service, having DSL was related to better results on comprehension of communication, compared to HL alone. This finding differs from that of a prior study which found that DSL is associated with greater communication difficulties compared to HL [21]. These contrasting results may arise from methodological discrepancies between our studies, specifically this prior study used a different measure of communication function to ours and whereas we assessed sensory impairment based on self-report, they did so using objective measures. Furthermore, their definition of VL was based on visual field restriction whereas our was based on difficulties with near vision.

With respect to depression we found a significant difference among sensory impairment groups in that *only* participants with VL, but none of those with HL or DSL, had scores that suggested clinical depression. This result is in line with a prior study that found that VL but not DSL is associated with depression [43]. It is noteworthy that these similar results come from two studies that used different measures of depression. Our finding on depression contradicts several other studies that found: no differences between DSL and single sensory impairment [35,36], worse results for DSL when compared to both VL and HL [38–41], and worse results for DSL only when compared to HL [37,42]. There are several differences between these prior studies and ours which may account for the mixed findings including diverse measures of sensory impairment and depression, and sample’s age range. The finding that our DSL participants were not worse off than those with VL only may be related to the fact that most participants with DSL had either minimal or moderate hearing difficulty. It is possible that having minimal to moderate issues with hearing does not impact a person’s risk of depression significantly more than as a result of VL alone. The worse findings in participants with VL, compared to DSL is likely not due to those with VL having worse visual functioning as participants in these two groups did not differ significantly with respect to most visual impairment characteristics.

Taken together, our findings on the comparison of assessment results of the three sensory groups support the idea that having DSL does not always equal worse health or functional status than having a single sensory impairment, and that the differential effects depend on the type of task [19]. In addition, our results suggest that the specific comparison group (whether VL or HL) also influences if DSL shows poorer outcomes.

When considering our findings, is important to keep in mind several limitations. Because our participants had received rehabilitation services at some point in the past, our results cannot be extended to older adults with sensory impairment who have not receive such services, or where they are not available. At the same time, our participants may be self-selected in ways that differentiate them from other members of the population of older adults receiving rehabilitation services. They were willing and able to take part in a study that required being interviewed with an extensive set of questions (about 300 items). This requires a level of stamina, independence and cognition that may be higher than those found of the rehabilitation centre clients who did not agree to participate in the study (e.g., those who may have other or additional health issues). Across all sensory impairment groups, a minority of participants had severe sensory difficulties, and a majority had minimal. These levels of sensory impairment may be below those of the clients who did not participate in this study. All participants had an initial evaluation by the centre dated at least 6 months prior to data collection. This excluded clients who are newer to receiving rehabilitation services, while it strengthens the possibility that the sample’s high functioning results arise in part from the effect of rehabilitation services. Participants were recruited irrespective of their age at sensory impairment onset with the final sample having most participants acquired sensory impairment at age 65 years or older. Our findings cannot be generalized to older adults whose age at sensory impairment onset are different (e.g., 85+ years) and face different challenges for adapting to the sensory loss (e.g., have had less time to adapt). It is also important to consider the limits of the interRAI CHA’s evaluation of health outcomes. For instance, this assessment identifies VL based on difficulties with near vision without considering issues with distance vision or contrast sensitivity. Similarly, it screens for difficulties with cognition that warrant further neuropsychological assessment but it does not diagnose specific cognitive conditions. Lastly, the study’s data is cross-sectional and does not allow to investigate whether and how the impact of sensory impairment changes over time.

In summary, our study contributes to the currently limited research that uses the interRAI CHA for the comprehensive assessment of older adults with DSL. Our findings suggest that among community-dwelling older adults with DSL, or single sensory impairment, who receive sensory rehabilitation services, a majority show high functioning in the areas of cognition, communication, ADL, IADL, depression and psycho-social well-being. Our findings support earlier research which indicated that, contrary to what could be expected, DSL is not always associated with worse results compared to single sensory impairment. Rather, the differential effects depend on the type of task and the specific comparison group (whether VL or HL). Further research is needed to better characterize older adults with DSL, particularly individuals with more severe sensory and cognitive difficulties than those in our sample, and in those who are not receiving rehabilitation services.

## Supporting information

S1 Table Scale CAP information. (DOCX)

S2 Table Additional CAP results. (DOCX)

S3 Table Sex associations. (DOCX)
