# Supplementary material for: Older adults with dual sensory loss in rehabilitation show high functioning and may fare better than those with single sensory loss
Source: PLoS One. 2020 Aug 3;15(8):e0237152. doi: 10.1371/journal.pone.0237152 (PMC7398548; doi:10.1371/journal.pone.0237152)
Supplement: S1 Table — (DOCX) [file pone.0237152.s001.docx]

**Table. Information about the interRAI CHA and DbS Health Index Scales and Clinical Assessment Protocols (CAP)**

| **Health Index Scale or CAP** | **Description** | **Scores** |
| --- | --- | --- |
| Activities of Daily Living Self-Performance Hierarchy Scale | Measures functional performance based on items regarding personal hygiene, locomotion, toilet use, and eating. This Scale is consistent with other measures of ADL [1]. | Scores can range from 0 (independent) to 6 (total dependence). We grouped the scores into 3 strata with increasing need for assistance:  (a) Independent (0).  (b) Supervision required (1), limited impairment (2).  (c) Extensive assistance required - 1 (3), extensive assistance required - 2 (4).  This grouping of scores was used in previous research [2]. |
| Cognitive Performance Scale | Assesses a person’s cognitive performance. It is based on 4 items:  1. Short term memory problems.  2. Cognitive decision making.  3. Expressive communication.  4. Independence in eating.  This Scale was validated against the Mini-Mental State examination [4], the Test for Severe Impairments [3] and the Montreal Cognitive Assessment [5]. | Scores can range from 0 (cognitively intact) to 6 (very severe impairment). We grouped the scores into 2 levels that differentiated between participants that were cognitively intact and those who were not:  (a) Intact (0).  (b) Borderline intact (1), mild impairment (2).  Scores 1 and 2 were combined because of their low frequencies (in all groups 91% or more of participants scored 0). |
| Deafblind Severity Index Scale (DbSI) | Identifies persons with Dual Sensory Loss (DSL). It is derived from two interRAI CHA items:  1. Ability to see close objects in adequate lighting, with visual device if used.  2. Ability to hear during past three days, with hearing device if used.  A higher DbSI score is associated with greater difficulties interacting with others and performing instrumental activities of daily living [6]. | Scores can range from 0 (no impairment in either sense) to 5 (severe impairment in both senses). A score of 3 (both senses mild/moderately impaired) or more indicates the presence of DSL.  We report the number of participants that scored 3 or more, that is, the number of participants that had DSL. |
| Depression Rating Scale (DRS) | Screens for symptoms of depression based on seven items related to mood and behaviour (e.g., persistent anger, unrealistic fears). Scores are based on the presence of a symptom and its frequency. A higher score does not necessarily imply more severe symptoms.  Research shows that a DRS score of 3 or higher is a valid indicator of clinical depression [9,10]. | Scores can range from 0 (symptoms not present) to 14 (symptoms exhibited daily in last 3 days).  Scores were grouped into two strata:  (a) No signs/symptoms of depression (score of 0-2).  (b) Signs/symptoms of depression (score of 3+). |
| Instrumental Activities of Daily Living (IADL) Capacity Scale | Summarizes difficulty with the IADLs:  1. Ordinary housework (e.g., doing dishes, laundry).  2. Meal preparation.  3. Phone use (with assistive devices if needed).  This scale is highly correlated with the Lawton IADL scale [7]. | Scores can range from 0 (independent in all tasks) to 6 (totally dependent or needing extensive or maximum assistance in all tasks).  We grouped the scores into 3 strata with increasing need for assistance:  (a) Independent (0).  (b) Set-up help only (1), supervision (2).  (c) Limited assistance (3), extensive assistance (4).  This grouping of scores was used in previous research [2]. |
| Pain Scale | Assesses the presence and intensity of pain based on two items:  1. Frequency of pain  2. Intensity of pain.  This scale is correlated with a gold standard clinical measure of pain, the Visual Analog Scale [8]. | Ranges from 0 (no pain) to 3 (severe daily pain).  We grouped the scores into 3 strata with increasing levels of pain intensity and frequency:  (a) No pain (0).  (b) Less than daily pain (1), daily not severe pain (2).  (c) Daily severe pain (3), daily excruciating pain (4). |
| Appropriate medication CAP | Identifies respondents at risk for an adverse drug effect.  It is based on the items: dizziness or lightheadedness, chest pain, edema, shortness of breath, poor self-rated health, dose, route of administration and frequency of taking medications, change in self-sufficiency (includes self-care performance and support, continence patterns, etc.) | 0 = not triggered. 1 = triggered – high priority. |
| Cognitive CAP | Focuses on helping persons with reasonable cognitive skills to remain as independent as possible. It is calculated based on the Cognitive Performance Scale and the items: episodes of disorganized speech, mental function varies, acute change in mental status, worsening decision making, understood, understand, repetitive complaints, something terrible about to happen, wandering, physically abusive, Alzheimer's dementia, other dementia, less than six months to live, overall change in care needs. | 0 = not triggered.  1 = triggered to prevent, indicating that the respondent has early indications of cognitive problems and is at a higher risk of declining further in the near future. |
| Communication CAP | Identifies between respondents whose communication functioning could be improved with intervention, and those that require a plan to prevent further decline in communication.  It is based on one cognitive item (decision making) and two communication items (expression and comprehension). | 0 = not triggered.  1 = respondent’s communication functioning could be improved with intervention.  2 = respondent requires a plan to prevent further decline in communication. |
| Dehydration CAP | Identifies persons who are dehydrated or have insufficient fluid intake.  It is based on the items: easily distracted, episodes of disorganized speech, mental function varies, acute change in mental status, dizziness, no bowel movement in 3 days, diarrhea, vomiting, fever, unintended weight loss, fluid intake <1,000 cc/day, dehydrated. | 0 = not triggered.  1 = triggered – low level.  2 = triggered – high level. |
| Informal Support CAP | Identifies persons who require help with instrumental activities of daily living, whose families are challenged to respond to these needs. It is based on the items: meal difficulty, housework difficulty, shopping difficulty, lives alone or in group setting, alone long periods of time, no primary helper. | 0 = not triggered.  1 = triggered indicating respondent needs help with 1 or more instrumental activities of daily living and shows at least 2 of the following: lives alone or in group setting, alone long periods of time, no primary helper. |
| Instrumental activities of daily living (IADL) CAP | Identifies persons with potential for improvement in IADLs. It is based on the following scales: IADL, Activities of Daily Living (ADL), Cognitive Performance, and the items: meal preparation difficulty, ordinary housework difficulty, shopping difficulty,  transportation difficulty, client believes he/she is capable of increasing independence, caregiver believes client is capable of increasing independence, ADL status has become worse. | 0 = not triggered.  1 = triggered – with potential  to improve. |
| Mood CAP | Identifies respondents that have a pre-existing diagnosis of depression or a depressed mood, and can be triggered indicating either medium or high risk for depression.  It is based on the Depression Rating Scale (DRS) which screens for symptoms of depression based on seven items related to mood and behaviour (e.g., persistent anger, unrealistic fears). The DRS cores range from 0 (symptoms not present) to 14 (symptoms exhibited daily in last 3 days). | 0 = not triggered (DRS score of 0).  1 = triggered - medium risk of depression (DRS score of 1 or 2).  2 = triggered - high risk of depression (DRS score of 3 or higher).  Research shows that a DRS score of 3 or higher is a valid indicator of clinical depression [9,10]. |
| DSL Orientation and Mobility (O&M) CAP | This CAP applies only to respondents with Dual Sensory Loss. It assesses the ability to move about independently in familiar and unfamiliar environments.  This CAP is based on the CPS, the DbSI, and three items regarding independent travel. | 0 = not triggered.  1 = triggered indicating difficulty with O&M in unfamiliar environment.  2 = triggered indicating difficulty with O&M in familiar environment. |
| Prevention CAP | Identifies people who may have poor preventive health care.  It is based in the items: influenza vaccine; pneumovax vaccine; mammogram; blood pressure; dental exam; hearing exam; eye exam;  colonoscopy; physician visit (count); physician visit (days); sex – female. | 0 = not triggered.  1 = triggered - respondent has had recent physician visit but has not followed one or more preventive measures (e.g.: influenza vaccine).  2 = triggered - respondent has not had recent physician and has not followed one or more preventive measures. |
| Social Relationship CAP | Identifies respondents with reduced social relationships that warrant interventions to facilitate social engagement.  It is based on the Cognitive Performance Scale and the items: understands, loneliness, change in social activities, length of time alone, failure to pursue life in facility. | 0 = not triggered  1 = triggered for care plan follow up |
| Urinary Incontinence CAP | Identifies respondents whose urinary function could improve, and those who could respond to a treatment program to prevent worsening of urinary function.  It is based on the items: decision making, walking, decline in activities of daily living, bladder continence, indwelling catheter, hip fracture, pneumonia, diarrhea, scheduled toileting program. | 0 = not triggered – continent at baseline.  1 = triggered – prevent decline.  2 = triggered – facilitate improvement. |

**References**

1. Morris J, Fries B, Morris S. Scaling ADLs with the MDS. J Greontology Med Sci. 1999;54A: M546–M553. doi:10.1093/gerona/54.11.M546

2. Guthrie DM, Declercq A, Finne-Soveri H, Fries BE, Hirdes J, Puts M, et al. The Health and Well-Being of Older Adults with Dual Sensory Impairment (DSI) in Four Countries. Chen K, editor. PLoS One. Public Library of Science; 2016;11: e0155073. doi:10.1371/journal.pone.0155073

3. Morris JN, Fries BE, Mehr DR, Hawes C, Phillips C, Mor V, et al. MDS Cognitive Performance Scale. J Gerontol. 1994;49: M174–M182. doi:https://doi.org/10.1093/geronj/49.4.M174

4. Hartmaier SL, Sloane PD, Guess HA, Koch GG, Mitchell CM, Phillips CD. Validation of the minimum data set cognitive performance scale: Agreement with the mini-mental state examination. J Gerontol Med Sci. 1995;50: M128–M133. doi:10.1093/gerona/50A.2.M128

5. Jones K, Perlman CM, Hirdes JP, Scott T. Screening Cognitive Performance With the Resident Performance Scale. La Rev Can Psychiatr. 2010;55: 736–740. Available: http://eutils.ncbi.nlm.nih.gov/entrez/eutils/elink.fcgi?dbfrom=pubmed&amp;id=21070702&amp;retmode=ref&amp;cmd=prlinks

6. Dalby DM, Hirdes J, Stolee P, Strong JG, Poss J, Tjam EY, et al. Development and psychometric properties of a standardized assessment for adults who are deaf-blind. J Vis Impair Blind. 2009;103: 7–16.

7. Landi F, Tua E, Onder G, Carrara B, Sgadari A, Rinaldi C, et al. Minimum Data Set for Home Care: a valid instrument to assess frail or older people living in the community. Med Care. 2000;38: 1184–90.

8. Fries BE, Simon SE, Morris JN, Flodstrom C, Bookstein FL. Pain in U.S. nursing homes: Validating a pain scale for the Minimum Data Set. Gerontologist. 2001;41: 173–179. Available: http://www.embase.com/search/results?subaction=viewrecord&from=export&id=L32295091%0Ahttp://sfx.library.uu.nl/utrecht?sid=EMBASE&issn=00169013&id=doi:&atitle=Pain+in+U.S.+nursing+homes%3A+Validating+a+pain+scale+for+the+Minimum+Data+Set&stitle=Gerontologi

9. Burrows A. Development of a minimum data set-based depression rating scale for use in nursing homes. Age Ageing. 2000;29: 165–172. doi:10.1093/ageing/29.2.165

10. Martin L, Poss JW, Hirdes JP, Jones RN, Stones MJ, Fries BE. Predictors of a new depression diagnosis among older adults admitted to complex continuing care: Implications for the depression rating scale (DRS). Age Ageing. 2008;37: 51–56. doi:10.1093/ageing/afm162
