# Supplementary material for: Older adults with dual sensory loss in rehabilitation show high functioning and may fare better than those with single sensory loss
Source: PLoS One. 2020 Aug 3;15(8):e0237152. doi: 10.1371/journal.pone.0237152 (PMC7398548; doi:10.1371/journal.pone.0237152)
Supplement: S2 Table — (DOCX) [file pone.0237152.s002.docx]

**Table. Results of additional interRAI CHA and DbS Clinical Assessment Protocols**

|  | **Vision loss only** | **Hearing loss only** | **Dual sensory impairment** |  | |  |
| --- | --- | --- | --- | --- | --- | --- |
|  | **N = 58** | **N = 69** | **N = 73** |  | |  |
| **Clinical Assessment Protocol (CAP)** | **% (N)** | | | **p-value** | |  |
| *Appropriate medication CAP* |  |  |  |  | |  |
| Not triggered (0) | 92.6 (25) | 100 (33) | 96.7 (29) | p = 0.38 | |  |
| Triggered – high priority (1) | 7.4 (2) | 0 (0) | 3.3 (1) |  |  |  |
| *Dehydration CAP* |  |  |  |  | |  |
| Not triggered (0) | 79.3 (46) | 100 (69) | 95.9 (70) | p = 0.86 | |  |
| Triggered – low level (1) | 8.6 (5) | 0 (0) | 2.7 (2) |  |  |  |
| Triggered – high level (2) | 12.1 (7) | 0 (0) | 1.4 (1) |  | |  |
| *Falls CAP* |  |  |  |  | |  |
| Not triggered (0) | 86.2 (50) | 92.8 (64) | 83.6 (61) | p = 0.62 | |  |
| Triggered – medium risk (1) | 12.1 (7) | 5.8 (4) | 16.4 (12) |  |  |  |
| Triggered – high risk (2) | 1.7 (1) | 1.5 (1) | 0 (0) |  |  |  |
| *Urinary Incontinence CAP* |  |  |  |  | |  |
| Not triggered – continent at baseline (0) | 81.0 (47) | 84.1 (58) | 79.5 (58) | p = 0.14 | |  |
| Triggered – prevent decline (1) | 13.8 (8) | 11.6 (8) | 12.3 (9) |  |  |  |
| Triggered – facilitate improvement (2) | 5.2 (3) | 4.4 (3) | 8.2 (6) |  |  |  |
|  |  | | | |  | |
