# Supplementary material for: Older adults with dual sensory loss in rehabilitation show high functioning and may fare better than those with single sensory loss
Source: PLoS One. 2020 Aug 3;15(8):e0237152. doi: 10.1371/journal.pone.0237152 (PMC7398548; doi:10.1371/journal.pone.0237152)
Supplement: S3 Table — (DOCX) [file pone.0237152.s003.docx]

**Table. Sex associations**

|  | **Male**  **N = 78** | | **Female**  **N = 122** | |  |
| --- | --- | --- | --- | --- | --- |
|  | **% (N)** | | | | **p-value** |
| *Item: length of time alone during the day* | | | | | |
| Less than one hour (0), one to two hours (1) | | 66.7 (52) | | 38.5 (47) | 0.003 |
| More than two hours but less than eight hours (2) | | 24.4 (19) | | 50 (61) |  |
| Eight hours or more (3) | | 8.8 (7) | | 11.5 (14) |  |
| *Pain Health Index Scale* | |  | |  |  |
| No pain (0) | | 52.6 (41) | | 32.8 (40 | 0.01 |
| Less than daily pain (1), daily but not severe pain (2) | | 35.9 (28) | | 61.5 (75) |  |
| Daily severe pain (3), daily excrutiating pain (4) | | 11.5 (9) | | 5.7 (7) |  |
| *Informal Support CAP* | |  | |  |  |
| Not triggered (0) | | 88.5 (69) | | 62.3 (76) | 0.001 |
| Triggered (1) | | 11.5 (9) | | 37.7 (46) |  |
